# Restoration of bilateral motor coordination from preserved agonist-antagonist coupling in amputation musculature

**DOI:** 10.1186/s12984-021-00829-z

**Published:** 2021-02-17

**Authors:** Tony Shu, Shan Shan Huang, Christopher Shallal, Hugh M. Herr

**Affiliations:** 1 MIT Media Lab, 75 Amherst St, Cambridge, MA 02139 USA; 2 Department of Mechanical Engineering, MIT, 77 Massachusetts Ave, Cambridge, MA 02139 USA; 3 Department of Biomedical Engineering, Johns Hopkins University, 720 Rutland Ave, Baltimore, MD 21205 USA; 4 Physical Medicine and Rehabilitation, Harvard Medical School, 25 Shattuck Street, Boston, MA 02115 USA

**Keywords:** Neuromuscular modeling, Myoelectric prosthesis, Neural control of movement, Agonist-antagonist myoneural interface

## Abstract

**Background:**

Neuroprosthetic devices controlled by persons with standard limb amputation often lack the dexterity of the physiological limb due to limitations of both the user’s ability to output accurate control signals and the control system’s ability to formulate dynamic trajectories from those signals. To restore full limb functionality to persons with amputation, it is necessary to first deduce and quantify the motor performance of the missing limbs, then meet these performance requirements through direct, volitional control of neuroprosthetic devices.

**Methods:**

We develop a neuromuscular modeling and optimization paradigm for the agonist-antagonist myoneural interface, a novel tissue architecture and neural interface for the control of myoelectric prostheses, that enables it to generate virtual joint trajectories coordinated with an intact biological joint at full physiologically-relevant movement bandwidth. In this investigation, a baseline of performance is first established in a population of non-amputee control subjects ($n = 8$). Then, a neuromuscular modeling and optimization technique is advanced that allows unilateral AMI amputation subjects ($n = 5$) and standard amputation subjects ($n = 4$) to generate virtual subtalar prosthetic joint kinematics using measured surface electromyography (sEMG) signals generated by musculature within the affected leg residuum.

**Results:**

Using their optimized neuromuscular subtalar models under blindfolded conditions with only proprioceptive feedback, AMI amputation subjects demonstrate bilateral subtalar coordination accuracy not significantly different from that of the non-amputee control group (Kolmogorov-Smirnov test, $P \ge 0.052$) while standard amputation subjects demonstrate significantly poorer performance (Kolmogorov-Smirnov test, $P < 0.001$).

**Conclusions:**

These results suggest that the absence of an intact biological joint does not necessarily remove the ability to produce neurophysical signals with sufficient information to reconstruct physiological movements. Further, the seamless manner in which virtual and intact biological joints are shown to coordinate reinforces the theory that desired movement trajectories are mentally formulated in an abstract task space which does not depend on physical limb configurations.

**Supplementary Information:**

The online version contains supplementary material available at 10.1186/s12984-021-00829-z.

## Background

While the mechanisms to achieve full embodiment and agency over a prosthetic limb remain elusive and difficult to define, one reasonable intermediate requirement is achieving parity between bionic and intact physiology in motor control tasks. Critical to this objective are technologies that enable a neural bi-directional efferent-afferent control between the peripheral nervous system and the external prosthesis [1–5]. In clinical studies, afferent cutaneous signaling from the external prosthesis has been shown to improve prosthetic controllability, gait mobility, upper-extremity arm functionality, confidence, and mental and physical acuity during activities of daily living [6–10]. However, this is but one feedback pathway available from the peripheral nervous system.

In addition to cutaneous afferents, muscle-tendon proprioception via muscle spindles and Golgi tendon organs enable afferent sensory information of joint position, velocity, and torque [11] - fundamental feedback signals required for fine motor control and joint stability [12, 13]. To specifically address proprioceptive afferent feedback from a limb prosthesis using mechanoneural transduction, the agonist-antagonist myoneural interface (AMI) was recently developed [14–22]. The AMI is a surgically-constructed tissue architecture and neural interface system designed to provide persons with amputation improved proprioception and neuroprosthetic control. The AMI comprises mechanically linking two muscles to create an agonist and antagonist muscle pair such that contraction of one produces corresponding stretch of the other [15]. Its efficacy as a mechanoneural transducer has been demonstrated in animal models, where afferent neural signals recorded from the antagonist were found to be proportional to the functional electrical stimulation of the agonist [16, 21]. For a person with a limb amputation using an external prosthesis, at least one AMI muscle pair is created for each external prosthetic joint degree of freedom to be controlled. In current implementations at the transtibial amputation level, two AMI muscle pairs are created, one for the prosthetic ankle joint and a second for the prosthetic subtalar joint [18, 19]. As a person with a transtibial AMI amputation moves their phantom ankle-foot complex, the ankle and subtalar AMI constructs move dynamically as agonist-antagonist pairs [19]. It is hypothesized that this dynamic configuration produces afferent neural signals from existing muscle spindles and Golgi tendon organs within the AMI muscle-tendon units, and that these signals enhance phantom joint movement sensations for both the ankle and subtalar joints. In one ideal control paradigm for free-space prosthetic movements, these afferent neural signals provide vivid proprioception of perceived ankle and subtalar phantom joints whose kinematics are perfectly tracked by a powered prosthesis [18].

Early studies showed that unilateral transtibial amputee subjects with AMIs were able to activate them with lower co-contraction throughout leisurely movements compared to amputee subjects without AMIs activating analogous residual musculature [18, 19]. Further, with surface electromyography (sEMG) measured from the skin adjacent each AMI muscle, Clites et al. demonstrated AMI-driven position and impedance control of an ankle-subtalar powered prosthesis in an ambulatory task across uneven terrain [18]. While promising, these early findings provided little insight into the *limitations* of the transtibial AMI’s dynamic control capabilities during both high-velocity and bilaterally coordinated movements. The objective of this study, then, is to determine if persons with a unilateral transtibial AMI amputation can reproduce the bandwidth and unconstrained movement characteristics demonstrated by non-amputees during bilateral joint coordination between the affected and unaffected limbs.

Currently, the definition of bandwidth as it applies to lower-extremity movements is not well-defined. Unlike electromechanical systems that are commonly assessed by their ability to track a desired sinusoidal set point or to reject disturbances, classical notions of servo bandwidth and loop gain are not directly applicable to nonlinear human systems. Previous characterizations of human movement, including Fitts’s law and its derivatives, have aimed to quantify the precision and accuracy of discrete upper-extremity reaching movements using visual targets [23], and the related study of these movements’ time-normalized velocity profiles has also elucidated unique human motor control tendencies such as those described by the minimum-jerk hypothesis [24–26]. However, one particular area of prior research that started with rhythmic bimanual coordination of the index fingers is more directly relevant to our goal of understanding the limitations of human ability in terms analagous to bandwidth. These studies, which investigate symmetric and parallel coordination patterns, have produced significant insights into the strategies underlying human motor control and trajectory planning that evolve with increasing movement frequency [27–30].

Previous studies on bimanual coordination found that movements performed in mirror symmetry are more stable than those performed in parallel: with increasing tempo, parallel patterns tend to involuntarily decay into symmetry patterns, though the converse does not occur [27–30]. Mechsner et al. determined that this tendency is not due to a preference toward activation of homologous muscles, but rather due to a bias toward symmetry of trajectories formulated using perceptual, task space variables [27]. Correspondingly, rhythmic coordination of ipsilateral hand and foot has been shown to be more stable in same-direction movements compared to opposite-direction movements, despite the vastly different limb structures involved [31, 32]. Together, the studies suggest that so long as motor function remains non-pathological, certain coordination tendencies remain invariant across the body.

Through consideration of rhythmic bilateral coordination patterns between left and right legs, we hypothesize that persons possessing the transtibial AMI can control and coordinate a virtual prosthetic subtalar joint in a manner indistinguishable from persons without amputation, whereas persons possessing a standard transtibial amputation lacking proprioceptive afferents cannot demonstrate movement patterns indistinguishable from persons without amputation. Here, we investigate the subtalar joint over the ankle joint due to its ability to coordinate both bilaterally symmetric and parallel movements.

**Fig. 1:**
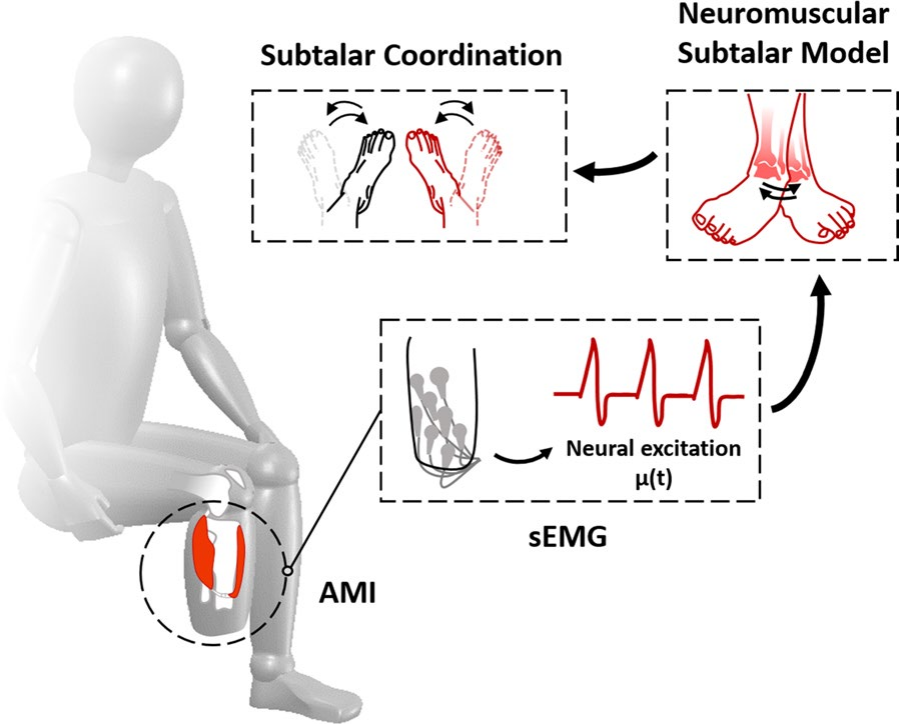
An AMI amputation subject generates phantom subtalar kinematics through a neuromuscular model. Neural excitation signals from agonist and antagonist muscles are processed from surface electromyography (sEMG) recorded during coordination tasks. These signals determine muscle activation in a modeled subtalar joint to generate subtalar kinematics

To evaluate this hypothesis, the first part of this study investigates the ability of eight non-amputee control subjects to rhythmically coordinate their subtalar joints in bilaterally symmetric and parallel patterns. We adapt upper-extremity coordination tasks to the lower-extremity domain to allow for quantification of kinematics and controllability using previously qualified metrics from literature [27–30]. The second part of our study investigates five unilateral AMI amputation subjects and four standard amputation subjects performing the same rhythmic bilateral subtalar coordination tasks as the non-amputee control group. For each subject, we implement a modeling and optimization technique that produces a neuromuscular model of an amputee subject’s phantom subtalar joint based on their mirrored joint kinematics, as shown in Fig. 1. Previous work involving unilateral upper- and lower-extremity amputation has investigated optimization of neuromuscular models using subject-specific reference trajectories and recorded sEMG, and though the results demonstrated the utility of controlled neuromuscular models, these studies did not compare the ability of subjects controlling the models to the native ability of non-amputee subjects in free-space [33–42]. In distinction to these previous investigations, this study compares amputee subjects’ motor control tendencies against non-amputee subjects’ tendencies in the physical domain by using the optimized neuromuscular models to generate subtalar virtual joint kinematics for the amputated side.

## Methods

### Study design

In this study, we investigate the ability of the AMI surgical paradigm to provide greater volitional control of residual musculature over traditional amputation techniques for the purposes of kinematic trajectory generation. The controlled laboratory experiments were designed to: (i) identify meaningful metrics to assess baseline ability to volitionally control physically intact lower extremities in free space; (ii) demonstrate the validity of associating activation of residual musculature to mirrored joint kinematics; and (iii) enable direct kinematic comparison between volitional control of residual musculature and volitional control of intact lower extremities.

### Subject recruitment

Eight non-amputee subjects were recruited for the control group, averaging 20 ± 1 years old, 1.69 ± 0.10 m, and 74 ± 13 kg (mean ± 1 SD) with an even male-female split. Non-amputee control subjects were only excluded if they self-reported physiological disorders which would affect their ability to control movement of their intact ankle-subtalar joints. Five persons with unilateral transtibial amputation possessing AMI constructs and four persons with standard unilateral amputation without AMI constructs were also recruited. Subjects with unilateral amputation were excluded if they did not rate at least a K3 Medicare Functional Level, were less than 12 months postoperative, had a residuum length less than 10 cm from the medial condyle, or self-reported physiological disorders which would affect their ability to control movement of their intact ankle-subtalar joints. Table 1 lists all subjects with amputation along with corresponding details. The AMI- subject label prefix indicates possession of AMI constructs while the ST- prefix indicates possession of a standard transtibial residuum. All amputee subjects were Caucasian, right-side dominant, and had normal or corrected-to-normal vision. Experiments were conducted with informed consent at the Massachusetts Institute of Technology (MIT) Media Laboratory under the approval of MIT Committee on the Use of Humans as Experimental Subjects (COUHES) protocol #1906898371.

**Table 1:** Summary of subjects recruited with unilateral transtibial amputation

ID	Sex	Age (years)	Height (m)	Mass (kg)	Side	Cause	Years Post-op
AMI-1	F	42	1.68	84.8	Left	Burn	1.5
AMI-2	M	55	1.73	70.0	Left	Trauma	3.1
AMI-3	M	43	1.81	77.4	Right	Thrombosis	1.9
AMI-4	M	53	1.83	99.7	Right	Trauma	1.5
AMI-5	F	49	1.68	81.6	Left	Trauma	1.0
ST-1	M	25	1.78	109.0	Left	Clubfoot	1.9
ST-2	F	62	1.70	80.0	Right	Trauma	3.0
ST-3	M	47	1.91	83.9	Right	Trauma	26.0
ST-4	M	44	1.77	74.8	Left	Trauma	6.4

### Bilateral coordination tasks

All subjects were instructed to move their ankle and subtalar joints (both intact and perceived phantom) in time with a metronome under several bilateral coordination conditions. Joint kinematics were recorded from intact joints, and surface electromyography (sEMG) signals were recorded from residual limbs (Additional file 1: Fig. S1).

#### Coordination conditions

**Fig. 2:**
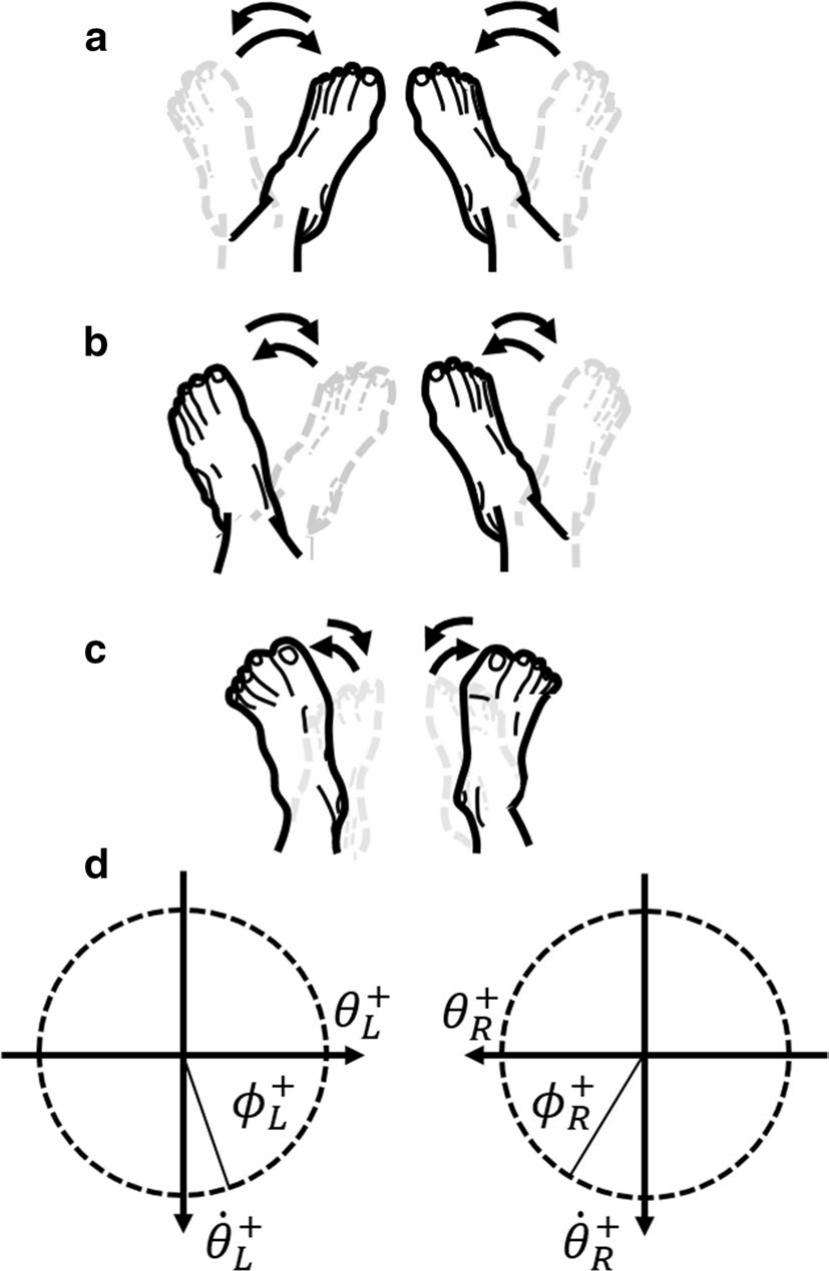
Bilateral coordination modes. **a** Coordinated subtalar joints under symmetry instruction. **b** Coordinated subtalar joints under parallel instruction. **c** Coordinated ankle joints under sagittal instruction. **d** Relative phase ($\theta _{rel}$) between two joints at any given point in time is defined as the difference in angles formed by their normalized velocities ($\dot{\theta }$) and normalized angular positions ($\theta$) during the movement according to Eqn. 3 [30]

The first set of binary coordination conditions addressed the presence of visual feedback. Tasks with vision required participants to look toward their feet while blind tasks were performed with eyes closed and blindfolded. The second set of conditions determined the patterns of ankle-subtalar movement. In the symmetric condition, subjects were instructed to fully invert both subtalars on the downbeat and fully evert both subtalars on the (silent) offbeat (Fig. 2a). In the parallel condition, subjects were instructed to fully evert the left subtalar and invert the right subtalar on the downbeat, mirroring the motion on the offbeat (Fig. 2b). In the sagittal condition, subjects were instructed to plantarflex both ankles on the downbeat and dorsiflex on the offbeat (Fig. 2c). All combinations of conditions with coded names are listed in Table 2.

**Table 2:** Bilateral coordination test conditions with coded names

	Symmetric	Parallel	Sagittal
Sighted	VisSym	VisPar	VisSag
Blind	BlindSym	BlindPar	BlindSag

Subjects completed three trials for each combination of conditions in random order, with a two-minute break between trials to limit effects from fatigue. Each trial required subjects to perform one cyclical motion per downbeat of a pre-recorded metronome audio track. Subjects with amputation were asked to volitionally activate their residual muscles as if to move their phantom joints. The minute-long audio track consisted of 5 sections with equal duration starting at a tempo of 1 Hz in the first section and incrementing by 0.4 Hz until a final tempo of 2.6 Hz (Additional files 2, 3, and 4).

Subjects were instructed to prioritize maintaining pace with the metronome track, sacrificing full range of motion of movements if necessary. Subjects were asked to repeat their first trial if they misunderstood the instructions (e.g., moving at half tempo). However, all subjects demonstrated a sense of rhythm and were observed to follow the tempo track to a reasonable degree. Inversion-eversion (IE) movements naturally involved some foot rotation in the transverse plane, though this degree of freedom was not explicitly measured. At the end of every trial, subjects performed a slow, controlled range of motion calibration test consisting of two dorsiflesion-plantarflexion (DP) cycles followed by two IE cycles to ensure invariant goniometer placement. Though subjects with amputation performed tests under sagittal instruction, the data are not reported here. All subjects were naive to the nature of the study .

#### Kinematics processing

Ankle-subtalar kinematics were collected from all subjects. An electromechanical relay was used to simultaneously trigger all related systems to synchronize each trial’s recorded data in time. A pair of commercial two-axis goniometers (Biometrics Ltd., Newport, UK) was used to measure all intact ankle-subtalar kinematics. Subjects were seated on the edge of a patient bed with knees at $90^{\circ }$ flexion. Subjects wore well-fitted athletic shoes. Goniometers were attached with one end adhered to the shoe heel and the other adhered to skin superior to the Achilles tendon. Goniometers were calibrated for subtalar axis and measurement axis parallelism by instructing subjects to perform slow, controlled subtalar motions while adjusting the positioning of the sensors. Afterward, goniometer ends were further secured to the shoe and shank using porous medical tape.

All ankle-subtalar position trajectories were recorded at a sampling frequency of 1,000 Hz. A 4th order 10 Hz low pass IIR Butterworth filter was applied forward and backward over the raw trajectory data. Velocity trajectories were generated from the filtered position trajectories by two-point forward finite differentiation.

#### Surface electromyography and signals processing

Surface electromyography signals were collected from the residual limbs of subjects with amputation. A commercial Refa (TMSi, Oldenzaal, Netherlands) 128-channel amplifier was used to collect sEMG signals. The skin was cleaned with isopropyl alcohol, and adhesive wet Ag/AgCl surface electrodes were placed in clusters to capture activity from the following residual muscles: tibialis anterior (TA), lateral gastrocnemius (LGAS), peroneus longus (PL), and tibialis posterior (TP). The residuum was palpated while asking subjects to perform ankle-subtalar movements to identify likely muscle locations. An additional ground reference electrode was placed on the patella of the affected side. 1.5 m shielded cables connected each electrode terminal to the Refa amplifier.

Monopolar sEMG signals were recorded at a sampling frequency of 2,048 Hz. From these, bipolar signals were reconstructed for each residual muscle after data collection based on the set of pairs which demonstrated the least amount of cocontraction during the calibration portion of the bilateral coordination tasks. A 4th order 10–500 Hz band pass infinite impulse response (IIR) Butterworth filter was designed in MATLAB R2019b (The MathWorks) and applied forward and backward over each recording. Filtered data were then rectified and normalized against the maximum voltage within each recording, assumed to represent maximum voluntary contraction. A 4th order 10 Hz low pass IIR Butterworth filter was then applied forward and backward to produce a record of neural excitation $\upmu$(t) [43]. Neural excitations were level shifted such that the minimum amplitude across each trial was 0.01 to avoid Thelen Hill-type muscle tendon unit (MTU) numerical singularities [44].

### Virtual subtalar modeling and optimization

A neuromuscular model was developed to produce a valid mapping from residual muscle activations to the physical domain of subtalar IE kinematics. The IE axis, as opposed to the DP axis, was chosen to facilitate comparison of bilaterally symmetric and parallel coordination between subjects with amputation and the non-amputee control group.

#### Neuromuscular subtalar model dynamics

**Fig. 3:**
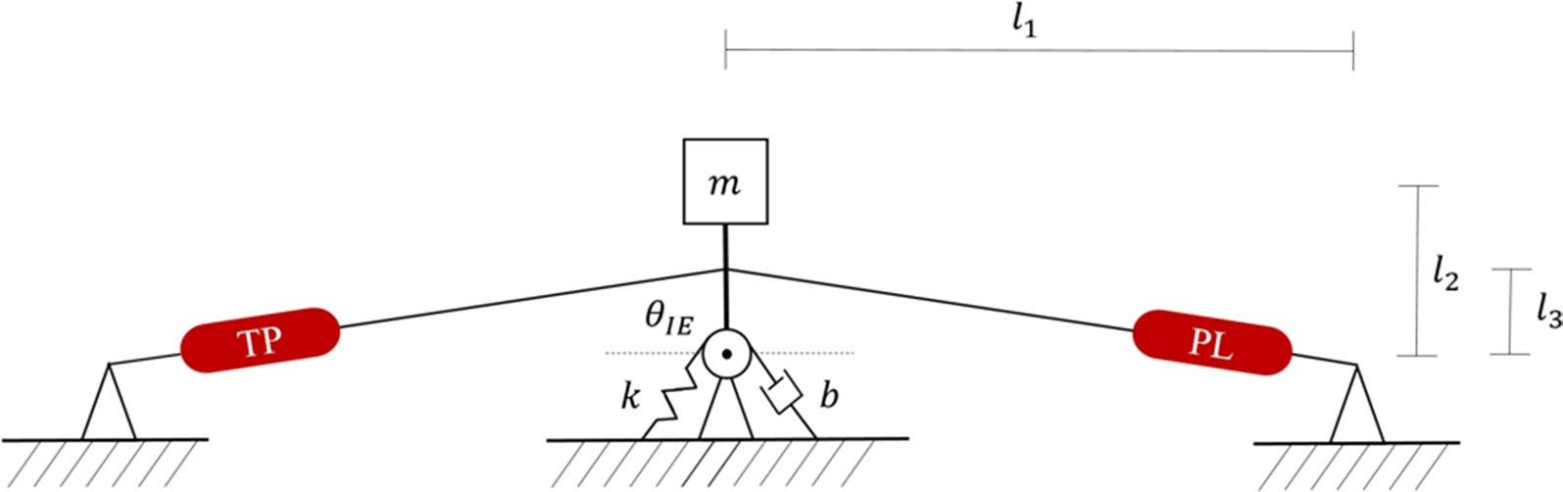
Neuromuscular subtalar model. Mechanical diagram of the base planar IE dynamic neuromuscular model with passive rotational stiffness (*k*), rotational damping (*b*), mass (*m*), and two Hill-type muscle-tendon units (MTUs) in agonist-antagonist configuration. Parameter value ranges are provided in Additional file 1: Table S1. Figure not drawn to scale

Consisting of only an agonist-antagonist muscle pair acting upon a second-order rotational mechanical system, the model is physically capable of generating modulated oscillatory kinematic trajectories from neural excitation inputs (Fig. 3). Importantly, this simple model was designed with deference to the idea that needless complexity obfuscates insight into underlying phenomena [45]. The model’s design does not replicate the exact morphology of an intact subtalar joint with accompanying tissue. Rather, it is specific enough to limit behavior to IE-like kinematics while retaining a sufficient number of tunable morphological parameters to fit a wide range of subjects. A bond graph representing detailed energetic relationships between model components is provided (Additional file 1: Fig. S2).

Referring to the dimensions of the model in Fig. 3, $l_{1}$ was chosen to be 0.7 *m*, equivalent to the horizontal span of each MTU. This is a relatively large value compared to other dimensions due to the finite stroke length of each MTU; the actuators need to be effective over the entire natural IE range of motion considering force generation limitations from optimal contractile element fascicle length and active force curve shape. A rotational domain was chosen over a linear one to allow effective MTU moment arms to vary over the range of motion in a physiologically relevant manner [46]. As $\theta _{IE}$ deviates from zero, effective MTU moment arm lengths decrease from the maximum value of $l_{3} = 0.1$ *m*, and the relative effectiveness of passive restoring stiffness *k*, modeled after ligament contributions, increases. Damping *b* is modeled to represent energetic losses from tissue sliding surfaces. Point mass $m = 0.4$ *kg* is located at a distance $l_{2} = 0.2$ *m* from the pivot and thus has a moment of inertia $J = 0.016$ ${kg \cdot m}^2$.

Including state variables for the MTUs, dynamics of the planar subtalar model are given by

1 $$\ddot{\Theta }_{IE} = J^{-1} [( F_{TP} ( \mu _{TP},l_{TP},v_{TP})  - F_{PL}( \mu _{PL},l_{PL},v_{PL})) \cdot l_3cos( \theta _{IE} ) - b\dot{\Theta }_{IE} - k\Theta _{IE} ]$$

where $F(\mu ,l,v)$ is the force generated by an MTU through the nonlinear dynamics of a Hill-type muscle model with $\mu$, *l*, and *v* being the excitation, length, and velocity of the MTU’s contractile element as a function of time. A thorough explanation of the specific Hill-type muscle model implemented is given by Thelen [44].

#### Genetic algorithm optimization

A genetic algorithm was used to optimize the model’s morphological parameters for each subject to minimize the residual sum of squares between the mirrored subtalar trajectory and the model’s output trajectory within a three-second window of training data. Because slow movements are difficult for humans to produce smoothly [47], and fast movements are difficult to produce accurately [23], the three-second window of training data was taken from the intermediate 1.4 Hz section of each subject’s blind symmetry trials to minimize trajectory error generated by the subject. From this window, raw sEMG data from residual inverter and everter muscles were processed using techniques described previously to produce estimated neural excitations $\mu (t)$. Mirrored IE trajectories from the contralateral intact subtalar were used as the desired reference trajectory.

The subtalar model was implemented in OpenSim 4.0, an open source dynamics environment widely used for neuromuscular modeling [48, 49]. MATLAB’s genetic algorithm (GA) toolbox was used to find the global optimum values of the MTU morphological and dynamic model parameters (Additional file 1: Table S1). MTU variables which belong to OpenSim’s Thelen2003Muscle class not explicitly mentioned here were fixed to their default values. A total of 18 MTU and 2 model parameters were investigated over the ranges specified in the table. The optimal parameter set minimized the residual sum of squares between generated trajectory and reference trajectory using the single objective cost function

2 $$\begin{aligned} C\left( \theta \left( t \right) ,\theta _{ref}\left( t \right) \right) = 1 - R_{\theta }^2 = \frac{\sum _{t=0}^{n-1}\left( \theta (t)-\theta _{ref}(t) \right) ^2}{\sum _{t=0}^{n-1}\left( \theta _{ref}(t) - \bar{\theta }_{ref} \right) ^2} \end{aligned}$$

where *t* is the discretized time index for trajectories *n* ms long. A full process diagram is provided (Additional file 1: Fig. S3).

Every GA run was uniformly initialized across the solution space with 5,000 members and set to run for 100 generations or until stalling for 3 generations. Two elite members were preserved per generation. Each iteration within the GA performed an OpenSim forward simulation with the specified AMI neural excitation inputs into a neuromuscular subtalar model initialized to a specific population member’s parameters and the initial position of the reference trajectory. Three separate GA runs were performed for each subject, each taking approximately 12 hours on an AMD $\hbox {Ryzen}^{\mathrm{TM}}$ 2950X CPU with 16 parallel processing threads. The optimized parameter set from the three runs with the best performance was used for all subsequent analysis per subject.

### Relative phase analysis

The relative phase between bilateral joint movements may elucidate limitations of the human neuromuscular system related to volitional command bandwidth. The metric is insensitive to the absolute magnitudes of joint position and velocity, but is sensitive to the relative timing of agonist and antagonist activation. Thus, we interpret it as an indicator of temporal coherence of efferent motor commands descending from the central nervous system to the lower extremities.

Plantarflexion and inversion are treated as positive directions for their respective rotational axes. For each half cycle of joint movement considered, relative phase is calculated by noting the time index of the right side joint at its downbeat inflexion point (either maximum inversion or maximum plantarflexion) and calculating the phase of the left joint. If both joints remain perfectly coherent during sagittal or symmetric conditions, relative phase is $0^{\circ }$ throughout the movement. If both are perfectly parallel, relative phase is $180^{\circ }$. Negative relative phase values for either condition imply a lagging left joint while positive values imply a leading left joint. A generalized formula for relative phase is given as

3 $$\begin{aligned} \theta _{rel} = \phi _{R} - \phi _{L} = tan^{-1}\left( \frac{\dot{\theta _{R}}}{\theta _{R}} \right) - tan^{-1}\left( \frac{\dot{\theta _{L}}}{\theta _{L}} \right) \end{aligned}$$

where $\theta _{R}$ and $\theta _{L}$ are normalized angular positions and $\theta _{R}'$ and $\theta _{L}'$ are their respective normalized angular velocities for each half cycle (Fig. 2d). A relative phase of $0^{\circ } \pm 60$ is considered symmetric and $180^{\circ } \pm 60$ as parallel with anything in between an intermediate mode [27].

Half cycles for analysis were taken from a continuous portion of each movement frequency band starting 2 *s* after each tempo transition. Seven half cycles were analyzed per frequency band, per trial. With 5 frequency bands per trial and 3 trials per condition, each subject contributed 105 half cycles for analysis per condition. Relative phase measurements were binned into containers $15^{\circ }$ in width, normalized by the number of all samples within a frequency band, then plotted as surfaces to visualize distribution.

For subjects with amputation, relative phase plots for BlindSym and BlindPar conditions were generated using the intact subtalar and optimized modeled subtalar with corresponding excitations from all associated trials.

### Kinematic analysis

Time-normalized velocity profiles of discrete movements have traditionally been studied to identify characteristics of human motion independent of movement distance and movement duration [24–26, 50, 51]. Thus, in contrast to our previous analysis of volitional command bandwidth through relative phase, the analysis of time-normalized subtalar eversion to inversion velocity profiles may elucidate limitations of the human neuromuscular system related to mechanical consistency. By not distinguishing between symmetric and parallel coordination modes, thus disregarding any temporal and high level decision error from choosing between performing subtalar inversion or eversion, kinematic patterns observed across populations and frequencies may more closely relate to the way generalized movements are realized physiologically through lower level muscle synergies and individual muscle activations.

Average time-normalized velocity profiles from maximum eversion to maximum inversion were calculated from the non-amputee group’s right subtalar movements under blind symmetry instruction at 1.4 Hz and 2.2 Hz. Average time-normalized velocity profiles generated by subjects with amputation were calculated for both intact subtalar and optimized subtalar model outputs under the same conditions. Moderately slow 1.4 Hz and moderately fast 2.2 Hz subtalar trajectories were selected due to their significantly different relative phase distributions. The beginning and end indices for each individual movement were determined by detecting when speed rose above and fell below 3% of the maximum movement speed.

Additionally, lognormal distributions with bounded support (LGNB) have been shown to accurately describe human-generated velocity profiles from the ankle, subtalar, and wrist [50–52]. A LGNB distribution described by

4 $$\begin{aligned} V_{\sigma }(t) = \frac{D(t_1-t_0)}{\sigma \sqrt{2\pi }(t-t_0)(t_1-t)}exp\left\{ \frac{-1}{2\sigma ^2}\left[ ln\frac{(t-t_0)}{(t_1-t)}-\mu \right] ^2 \right\} \end{aligned}$$

was fitted to each average velocity profile using an iterative least squares curve fit solver, where *D* is total displacement of the movement, $t_{0}$ the time of the impulse command, $t_{1}$ the end time of the movement, $\mu$ and $\sigma ^{2}$ the mean and variance of $ln(t-t_{0})$. A thorough description of this equation as it applies to human movement is given by Plamondon et al. [50]. Coefficients of determination were calculated for the generated LGNB velocity profiles. Standard deviations of the average velocity profiles were calculated and graphed per normalized time point. Further reading about these methods, including velocity profile rejection criteria, can be found in a study on discrete volitional ankle-subtalar movements by Michmizos et al. [52].

### Statistical analysis

Relative phase distributions of modes other than the one instructed were compared within subject groups using paired two-tailed Student’s t-tests at a significance level of $\alpha = 0.05$. Relative phases within each mode (symmetry, parallel, and intermediate) were approximately normally distributed for each test condition by visual inspection. Comparisons between subject groups were made using unpaired two-tailed Student’s t-tests at a significance level of $\alpha = 0.05$. Empirical distributions of relative phase were compared between subject groups using the Kolmogorov-Smirnov test at a significance level of $\alpha = 0.05$. Shaded regions on empirical distributions of relative phase denote 95% confidence interval using Greenwood’s formula. For comparisons involving variance of time-normalized subtalar velocity profiles between groups, subtalar type, and movement frequencies, Bartlett’s test was used at a significance level of $\alpha = 0.05$. Time-normalized subtalar velocity profiles were graphed as mean ± 1 SD. All statistical analysis was performed in MATLAB R2019b (The MathWorks).

## Results

### Lower-extremity bilateral coordination shares rhythmic motor control tendencies with upper-extremity bilateral coordination

**Fig. 4:**
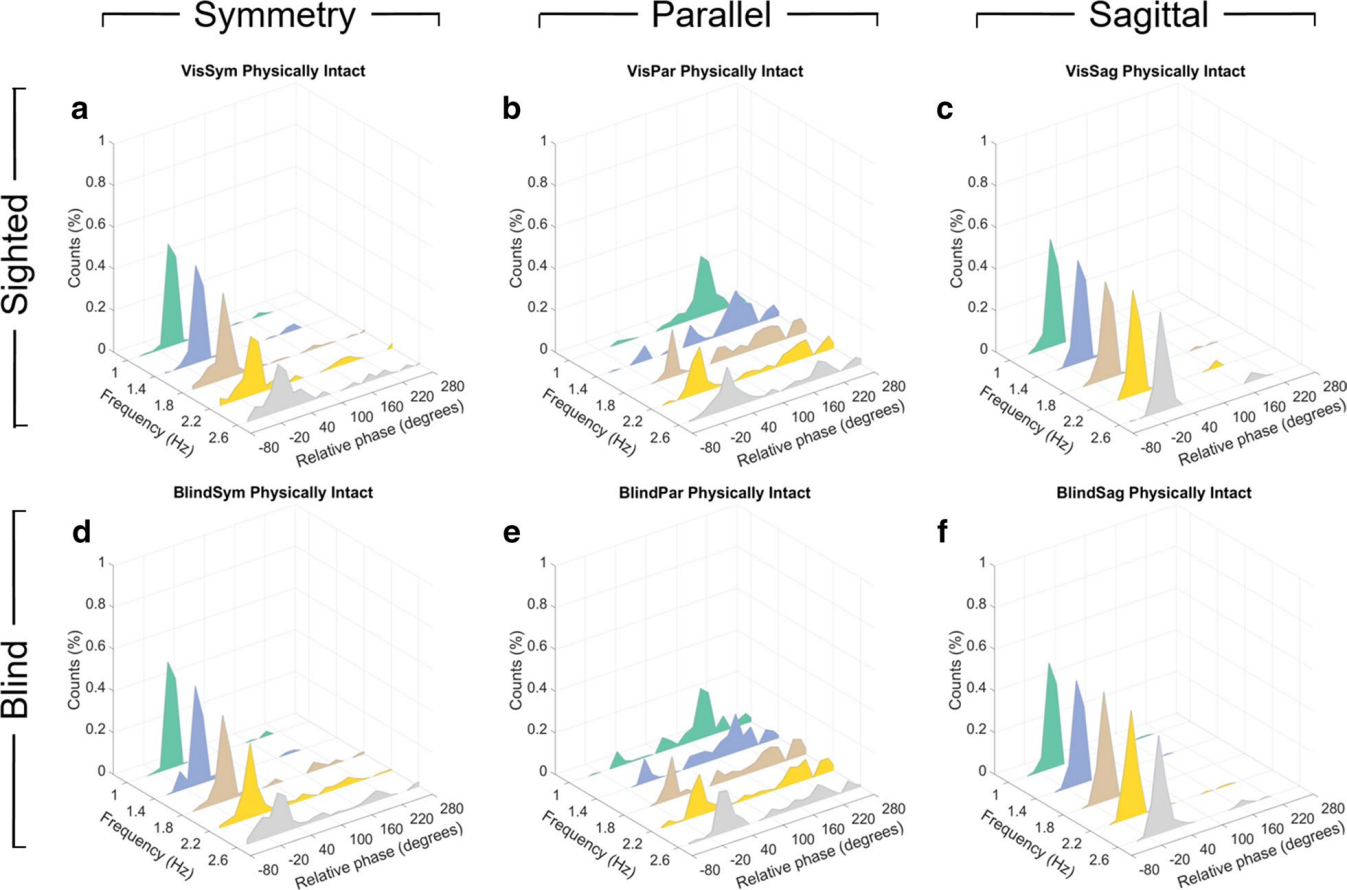
Bilateral coordination results for non-amputee control subjects. **a**–**c** Relative phase distributions of all sighted test coordination conditions (*n = 840* relative phase samples per subfigure). **d**–**f** Relative phase distributions of all blind test coordination conditions (*n = 840* relative phase samples per subfigure)

Non-amputee control subjects were not found to coordinate significantly differently in the sighted condition compared to the blindfolded condition (Fig. 4) when comparing distribution of modes other than the one instructed (paired t-test, $P = 0.967, P = 0.252, P = 0.543$ for symmetry, parallel, and sagittal conditions respectively), indicating subjects were able to perform just as well using only proprioceptive information. These results agree with previous literature on bimanual coordination and rhythmic reaching, suggesting that lower-extremity movements may also be formulated in dimensionally-reduced, perceptive task space variables which do not rely on vision [27].

Similar to Mechsner et al.'s bimanual coordination results [27], symmetric coordination of IE was found to be more stable than parallel in the non-amputee control group by measure of relative phase. Across all non-amputee subjects’ blind symmetry and blind parallel results (Fig. 4d, e), the proportion of modes other than the one instructed was 45.96% under parallel instruction while only 15.03% under symmetry, showing significantly higher deviation under parallel instruction (paired t-test, $P < 0.001$). Graphically, this is understood as the increasing accumulation of symmetric movement with frequency under blind parallel instruction and maintenance of symmetry dominance under blind symmetry instruction.

Compared to bimanual coordination figures from Mechsner et al.’s study [27], a greater occurrence of intermediate modes can be qualitatively observed for all tested IE conditions (Fig. 4a, b, d, e). Notably, symmetric coordination of DP under sagittal instruction showed significantly greater stability than symmetric coordination of IE (paired t-test, $P < 0.001$), with only 1.9% of movements deviating from symmetry.

### An optimized neuromuscular subtalar model generates mirrored joint kinematics

After demonstrating significant evidence of a unified motor control strategy across the entire body, one that: (i) plans trajectories in task-space coordinates independently of the biomechanical structures involved; (ii) does not depend on visual information; and (iii) is more stable performing symmetric motions, we proceeded to investigate the following claim for subjects with unilateral amputation: intact limb kinematics can be directly correlated with residual musculature sEMG during blind, bilaterally symmetric coordination tasks as subjects move their phantom joint to mirror their intact limb.

**Fig. 5:**
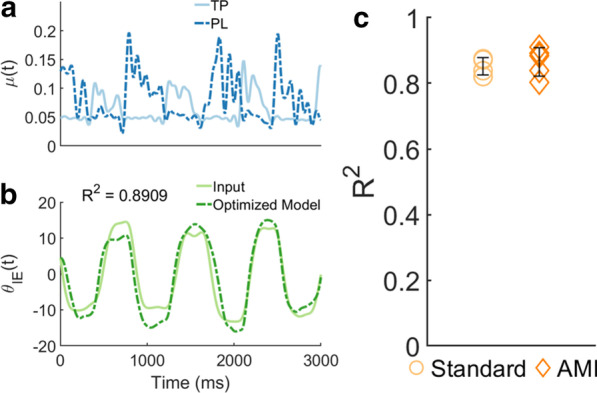
**a** Representative input neural excitations ($\mu (t)$) for the subtalar model’s tibialis posterior (TP) and peroneus longus (PL) muscles processed using measured sEMG from AMI-4’s (Additional file 4: Table S1) residuum for model parameter optimization. **b** Corresponding 1.4 Hz reference trajectory measured from AMI-4’s intact subtalar and optimized model output trajectory generated from input $\mu (t)$. Positive and negative values of $\theta _{IE}$ indicate inversion and eversion, respectively. **c** Optimized models’ coefficients of determination between reference and output trajectories average 0.851 ± 0.026 (mean ± 1 SD) for standard amputation subjects ($n = 4$) and 0.866 ± 0.044 for AMI amputation subjects ($n = 5$), indicating no significant difference in fit (unpaired t-test, $P = 0.5569$)

Utilizing the neural excitations processed from the residuum’s recorded sEMG as model input (Fig. 5a), the optimized models for both standard and AMI amputation subjects demonstrated a statistically similar (unpaired t-test, $P = 0.5569$), high degree of fit to the training data (Fig. 5c). A representative graph of reference input and optimized output IE trajectories shows high phase agreement with the majority of error due to range of motion differences (Fig. 5b).

### The agonist-antagonist myoneural interface achieves bilateral coordination indistinguishable from intact physiology

**Fig. 6:**
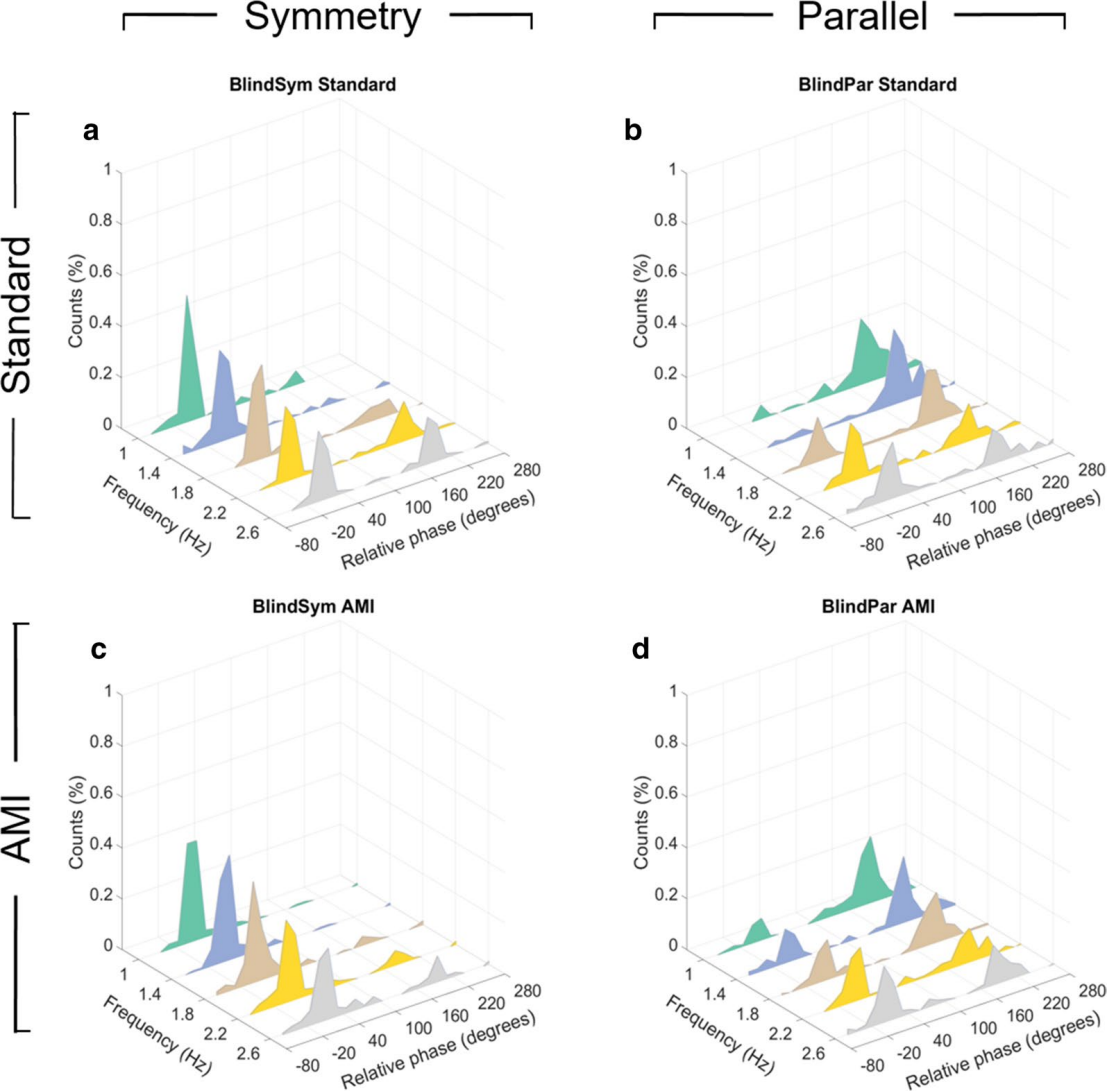
Bilateral coordination results between intact subtalar and optimized subtalar model for standard and AMI amputation subjects. **a,b** Relative phase distributions for standard amputation subjects under blind symmetry and parallel instructions ($n = 420$ relative phase samples per subfigure). **c,d** Relative phase distributions for AMI amputation subjects under blind symmetry and parallel instructions ($n = 525$ relative phase samples per subfigure)

After all amputee subjects’ subtalar models were optimized using training data, neural excitations processed using recorded sEMG data from all blind symmetry and parallel trials were then input into each corresponding subject’s optimized subtalar model to generate phantom subtalar kinematics. Relative phase metrics were calculated between intact subtalar and modeled subtalar kinematics for all amputee subjects (Fig. 6).

By inspection, it is clear both standard and AMI amputation subjects demonstrated instability under blind parallel instruction (Fig. 6b, d) with a greater tendency toward symmetrical coordination evolving with increasing tempo for both groups. Despite the vastly different biomechanical structures involved, this trend is qualitatively similar to that of the non-amputee control population (Fig. 4e), which is in turn similar to previously mentioned results from Mechsner et al. bimanual coordination studies [27]. However, AMI amputation subjects performed significantly better than standard amputation subjects under blind symmetry instruction (Kolmogorov-Smirnov test, $P = 0.021$) with the proportion of modes other than symmetry at 27.04% for standard amputation subjects (Fig. 6a) compared to only 14.52% for AMI amputation subjects (Fig. 6c). Further analysis reveals that the difference in proportion of modes other than the one instructed was statistically insignificant for standard amputation subjects between blind symmetry and blind parallel instructions at 2.6 Hz (paired t-test, $P = 0.053$). The cause of the insignificance can be attributed to standard amputation subjects deviating from symmetry coordination while under symmetry instruction at the fastest tested frequency (Fig. 6a). This is a tendency toward incoherency that has not been previously observed in extant work on limb coordination.

**Fig. 7:**
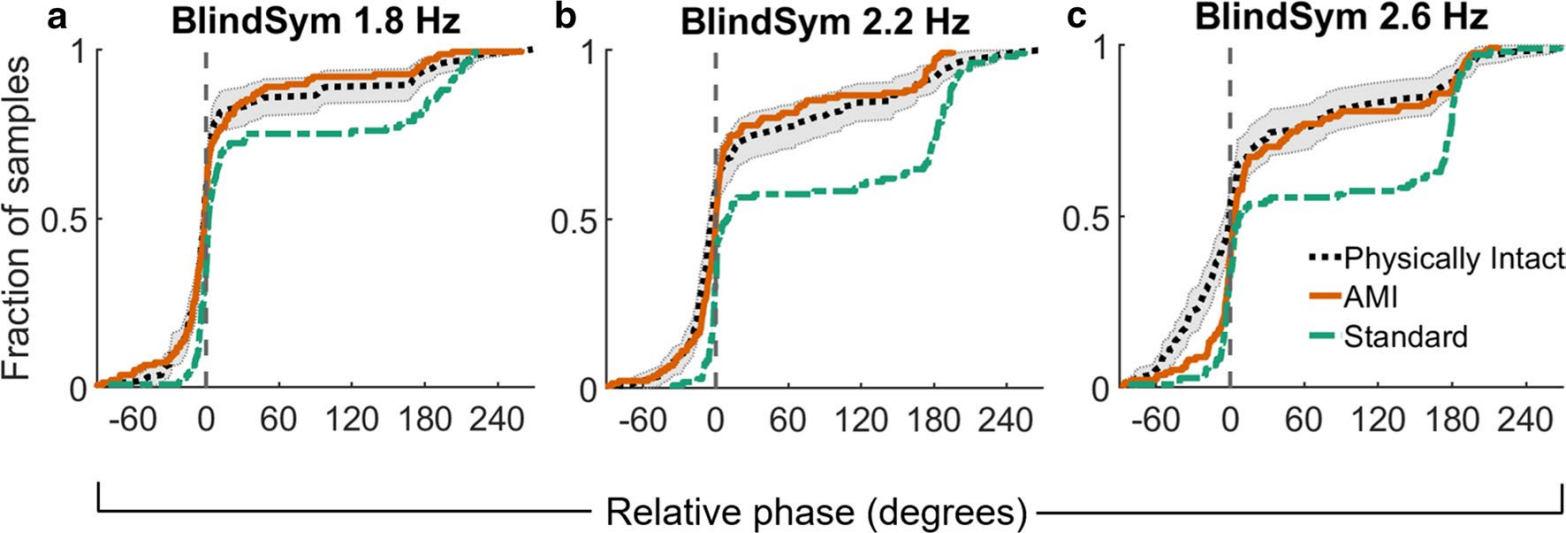
Empirical distribution functions of relative phase for **a** 1.8 Hz, **b** 2.2 hz, and **c** 2.6 Hz frequencies tested under blind symmetry instruction for all three subject groups. Shaded regions denote 95% confidence interval calculated using Greenwood’s formula. Vertical dashed gray line indicates $0^{\circ }$, or perfect symmetry

Additionally, by studying the empirical distribution functions of relative phase across all three subject groups under blind symmetry instruction (Fig. 7), it appears that AMI amputation subjects performed as well as control subjects in this task at the highest and most difficult movement frequencies tested. While the empirical distributions of relative phase generated by standard amputation subjects did not match those generated by the control subjects at 1.8 Hz, 2.2 Hz, and 2.6 Hz (Kolmogorov-Smirnov test, $P< 0.001, P< 0.001, P < 0.001$, respectively), the AMI amputation subjects demonstrated distributions indistinguishable from the non-amputee group at 1.8 Hz and 2.2 Hz (Kolmogorov-Smirnov test, $P = 0.94, P = 0.11$, respectively). At 2.6 Hz, AMI amputation subjects did produce relative phase distributions distinguishable from the control subjects (Kolmogorov-Smirnov test, $P = 0.001$). However, it is clear from their 2.6 Hz empirical distribution functions (Fig. 7c) that AMI amputation subjects were in fact more accurate than control subjects in achieving symmetry coordination, as seen by the tighter distribution of modes between $-\,60$ and $0^{\circ }$. A slight adjustment for this difference provides a more nuanced understanding of the subjects’ relative performance. Comparing 2.6 Hz relative phase distributions after shifting and treating all subjects’ symmetry mode movements from $-\,60$ to $0^{\circ }$ as perfect $0^{\circ }$ movements results in statistically indistinguishable distributions between AMI amputation subjects and control subjects (Kolmogorov-Smirnov test, $P = 0.052$). The same treatment applied to standard amputation subjects’ relative phases at 2.6 Hz still results in a distribution distinguishable from that of control subjects (Kolmogorov-Smirnov test, $P < 0.001$).

### The agonist-antagonist myoneural interface generates joint kinematics similar to intact physiology

If indeed the optimized subtalar models are able to accurately transfer relevant muscle activations to intended task space trajectories, there should be no significant differences between intact and modeled subtalar velocity profiles.

**Fig. 8:**
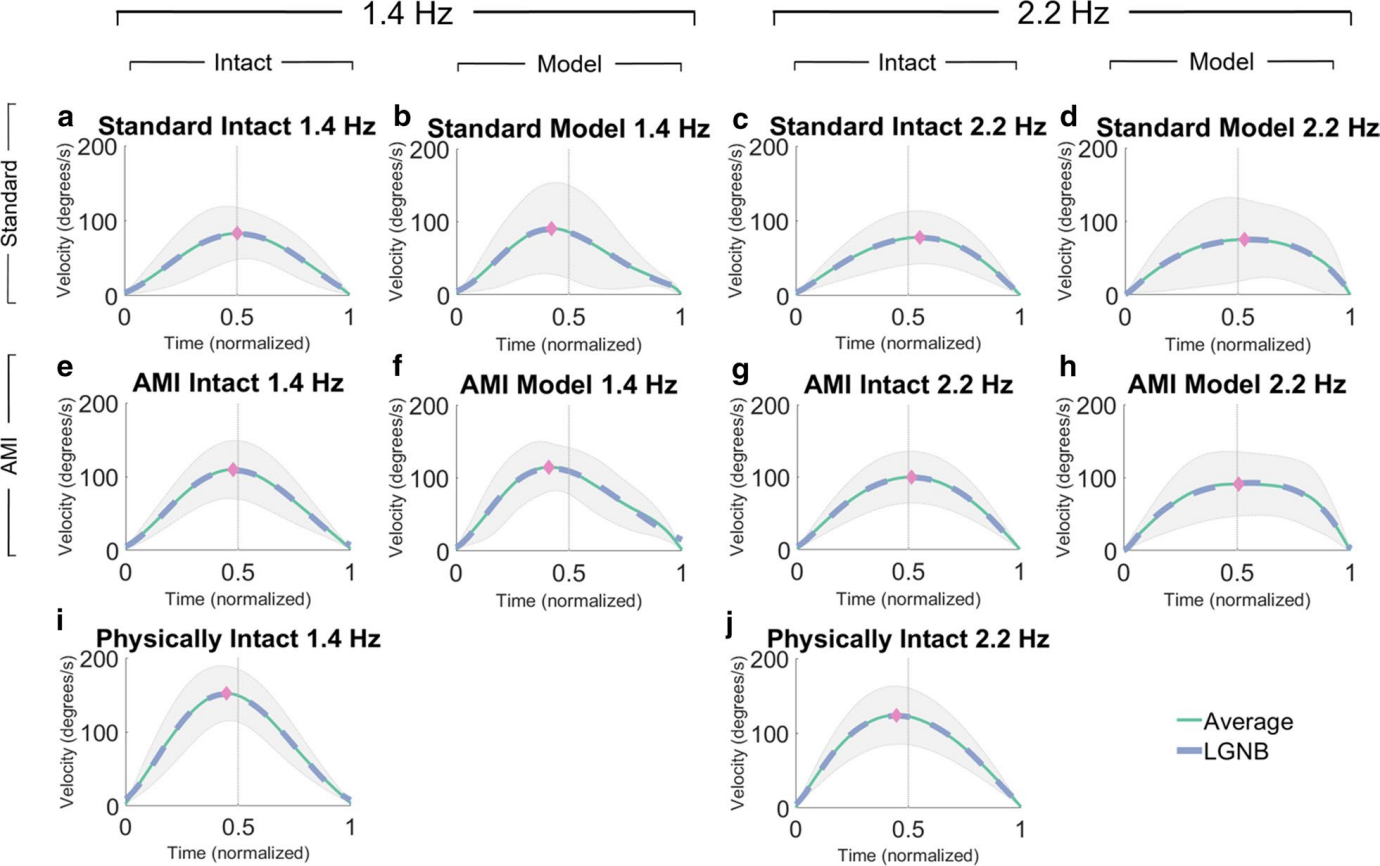
Time-normalized eversion to inversion subtalar velocity profiles under blind symmetry instruction from all subject groups. Velocity profiles were procedurally rejected from analysis if they possessed more than a single peak, indicative of a compound movement as proposed by Michmizos et al. [52] **a**–**d** Average time-normalized velocity profiles generated by standard amputation subjects. ($n \ge 29$ profiles per subfigure). Each subfigure contains profiles generated from both left and right sides. **e**–**h** Average time-normalized velocity profiles generated by AMI amputation subjects. ($n \ge 35$ profiles per subfigure). Each subfigure contains profiles generated from both left and right sides. **i**–**j** Average time-normalized velocity profiles generated by non-amputee subjects ($n \ge 45$ profiles per subfigure). Each subfigure contains profiles generated from the right side only

For movements of a given frequency, we interpret variance at maximum average velocity as one metric for consistency. By this metric, the standard amputation group’s modeled subtalar velocity profiles were less consistent compared to all others. All 1.4 Hz subtalar velocity profiles, excluding the standard amputation group’s modeled subtalar velocity profiles, have statistically equal variance at maximum average velocity when compared together (Bartlett test, $P = 0.707$) (Fig. 8a, e, f, i). This can be visualized by comparing the widths of the ±1 SD shaded regions at the indices of maximum average velocity designated by the pink diamonds. Substituting the standard amputation group’s modeled 1.4 Hz profiles (Fig. 8b) for the AMI amputation group’s profiles (Fig. 8f) and performing the same comparison results in significantly different variance at maximum velocity between groups (Bartlett test, $P < 0.001$). Qualitatively greater variance for the standard amputation group’s modeled 1.4 Hz profiles overall can be seen by the relatively larger area of the shaded region throughout the movement (Fig. 8b). Additionally, all 2.2 Hz subtalar velocity profiles, excluding the standard amputation group’s modeled subtalar velocity profiles, demonstrate statistically similar variance at maximum average velocity when compared together (Bartlett test, $P = 0.379$) (Fig. 8c, g, h, j). Substituting the standard amputation group’s modeled 2.2 Hz profiles (Fig. 8d) for the AMI amputation group’s modeled 2.2 Hz profiles (Fig. 8h) in the comparison results in significantly different variance at maximum velocity between groups (Bartlett test, $P = 0.005$). Thus, by this metric of variance at maximum average velocity, it is possible to distinguish movements generated by the subtalar models controlled by standard amputation subjects from intact subtalar movements, but it is not possible to distinguish movements generated by the subtalar models controlled by AMI amputation subjects.

Notably, despite the significantly different distributions of coordination modes at the highest movement frequencies tested (Fig. 7), variance at maximum average velocity does not differ significantly between intact subtalar velocity profiles at both 1.4 Hz and 2.2 Hz from all subjects (Bartlett test, $P = 0.927$) (Fig. 8a, c, e, g, i, j).

To relate these data to previous studies on time-normalized velocity profiles, a LGNB distribution was fitted to each average time-normalized velocity profile using nonlinear parameter optimization. Here, all average time-normalized velocity profiles are well-described by LGNB distributions ($R^2 \ge 0.994$), suggesting failure to reject any as non-humanlike. Skewness and kurtosis were also calculated for all individual velocity profiles (Additional file 1: Tables S2 and S3).

## Discussion

In this study, we demonstrate a modeling and optimization technique capable of producing phantom joint motion based on a fundamental understanding of human motor control. When the technique was applied to both unilateral transtibial amputees with the AMI and unilateral transtibial amputees with standard amputation architecture, a distinction in performance was found between the two subject groups. Standard amputation subjects were less coordinated and less consistent in their movements involving intact and modeled subtalar joints compared to both AMI amputation and non-amputee subjects. Meanwhile, AMI amputation subjects demonstrated movement coordination and consistency indistinguishable from non-amputee subjects through the metrics considered, supporting the hypothesis that the AMI construct provides improved proprioception and control of phantom joints through the mechanism of agonist-antagonist stretch compared to subjects with standard amputation.

Non-amputee subjects demonstrated a tendency toward symmetrical lower-extremity coordination with increasing movement frequency in both sighted and blindfolded conditions (Fig. 4). This tendency, previously observed in upper-extremity coordination [27–30], is attributed by Mechsner et al. to the formulation of volitional movement in dimensionally-reduced, perceptive task space variables which do not rely on vision [27]. If indeed the formulation of volitional movement trajectories is invariant across the body, the relatively lower coherence of symmetrical subtalar coordination observed in this study (Fig. 4a, d) compared to symmetrical bimanual coordination observed in Mechsner et al. study may then be attributable to evolved differences in lower-extremity and upper-extremity functionality. It is widely-accepted that upper-extremity development is both phylogenetically more recent and more specialized for dexterous manipulation compared to lower-extremity development. Early electrostimulation studies also showed that the hands, fingers, and face generate the vast majority of afferent somatosensory signals while the feet and legs are proportionally underrepresented [53], an understanding popularly represented as the distorted sensory homunculus. Thus, even if subjects know how their joints should be positioned in task space at any given point while coordinating, their instantaneous estimations of joint positions may be erroneous due to lower afferent sensation resolution, in turn producing intermediate coordination modes from formulation of movement trajectories based on incorrect positional information. Considering also the similarly distorted motor homunculus, efferent lower-extremity motor commands may be less accurate than upper-extremity commands even with perfectly formulated movement trajectories.

However, the gross difference in cortical resources dedicated to upper and lower extremities does not clearly explain the increased coherence observed of ankle coordination over subtalar coordination (Fig. 4a, c, d, e). Instead, the difference in volitional symmetric coordination within this same task space may be attributed to the varying roles of IE and DP during bipedal locomotion. If forward motion in the sagittal direction is considered to contain all the task space variables of interest, such as walking speed, stride length, and slope adaptation [54], it follows that there would also be relatively greater motor cortical resources dedicated to volitional control of corresponding DP muscle groups to adjust these parameters. Indeed, recalling the inverted pendulum model of bipedal locomotion, foot rotation in the roll axis exists primarily to stabilize trunk roll through contact with the ground [55]. In observations of human subjects, researchers have found that mediolateral center of pressure is significantly more variable than anteroposterior center of pressure when subjects are separately visually disturbed and blinded during walking, concluding that mediolateral stabilization relies more on reflexive feedback compared to the passively stabilized anteroposterior direction [56, 57]. Thus, in our tests of volitional ankle and subtalar coordination, the difference in performance under the two symmetric conditions may be understood as reflecting the maximal degree of volitional control required of ankle and subtalar muscles during normal locomotive behaviors.

Coordination results from modeled subtalar joint motion highlight important factors involved in human motor control, especially as they relate to amputation from the perspective of motor program theory. Briefly, motor program theory emphasizes stereotyped motor control patterns executed in a descending hierarchy from the central nervous system, to muscle synergies, to individual muscle activations themselves. Within this framework, afferent sensory signals are not strictly necessary for producing movement, but they do inform and correct movements as they occur [58]. Motor program theory would suggest that standard amputation subjects were unable to match the performance of the non-amputee control group due to incorrect movement adjustments involving the affected limb’s musculature. While they may be able to plan correct coordination trajectories at a high level in perceptual task space variables, they could fail to realize them due to afferent mechanoreceptor signals incongruent with expected joint motion that yield erroneous muscle activations, especially as movement frequency and task difficulty increase. In contrast, the fact that the AMI amputation group demonstrated bilateral coordination equivalent to the non-amputee control group indicates preservation of the motor control hierarchy. Importantly, the acute novelty of the AMI structure (compared to the natural musculature of an intact hand or leg coordinating pair) having no detrimental effect on coordination ability is a feasible result considering Mechsner et al.'s bimanual coordination and others' ipsilateral limb coordination studies which suggest coordination ability does not depend on homologous musculature [27, 31, 32].

In addition to coordination, analysis of kinematic characteristics of time-normalized eversion to inversion velocity profiles reveals motor control abilities for both intact and amputated physiology. Based on similar variance at maximum average velocity observed across frequencies and relative phase distributions from all subjects’ intact subtalars (Fig. 7), we conclude that: (i) specific coordination mode does not significantly influence unilateral kinematic consistency; and (ii) coordination between an intact subtalar and an amputated residuum does not make the intact subtalar’s movements less consistent for both standard and AMI amputation groups. The results of our kinematic analysis also show the AMI amputation group generating modeled subtalar velocity profiles as consistent as those from all intact subtalars, leaving the standard amputation group’s less consistent modeled subtalar velocity profiles to implicate issues stemming from differences between the two groups.

Overall, these results provide strong evidence that the AMI construct is capable of greater volitional controllability than residual musculature from standard amputation techniques. By standardizing the neuromuscular subtalar model, the optimization paradigm, and the test conditions, the obvious remaining differentiator between the two amputee subject groups involves the mechanical structure of their musculature. Specifically, the AMI allows agonist contraction with correlated antagonist stretch to produce afferent proprioceptive signals analogous to those from volitional joint motion, a fundamental feedback paradigm required for fine motor control and joint stability [12, 13]. In contrast, residual muscles from standard amputation techniques are anchored distally to bone or other connective tissues for the purposes of limb shaping and padding [59]. In this configuration, any agonist stretch is performed against an effectively infinite mechanical impedance with no relevant antagonist stretch. These afferent signals, analogous to those produced when a limb encounters an immobile obstacle, are incongruent with joint motion and may confuse the subject when attempting the coordination tasks.

At a minimum, the model optimization technique is sufficient to capture native coordination tendencies: there is significant evidence of a valid mapping from the chosen signal inputs to the optimized model outputs that accurately describes ideal coordination and kinematic outcomes for AMI subjects. The predictive power of the optimized neuromuscular subtalar model is supported by its generalizability to higher movement frequencies. Despite being optimized using only 3 *s* of training data taken from 1.4 Hz inversion-eversion cycles, AMI subjects were able to demonstrate symmetrical subtalar coordination up to the 2.6 Hz movement frequency tested.

General issues related to modeling error limit the insights into the intricacies of human motor control from this study. With the understanding that no complex dynamical system may be fully described by a simplified model, it is possible there exists some transformation of measured sEMG to the domain of rotational kinematics which demonstrates that the traditional amputation architecture is able to coordinate better with intact joints compared to the AMI architecture. However, because the fixed model parameters are based upon an intact subtalar and foot complex, there is little reason to believe that the optimized subtalar model is obviously biased to produce an advantage in either amputee group. The neuromuscular subtalar model is neither a model of the AMI nor a model of standard amputation musculature, but rather a tool for their utilization much in the way that a pen facilitates writing. Still, the results of this study must be interpreted in the limited context of the singular technique explored.

Related to achieving natural human behaviors, a final consideration pertains to the possible control disparities between bilateral and unilateral movements. These experiments demonstrated bilateral movement stability under symmetry coordination, which does not necessarily guarantee equal movement fidelity of unilateral movements. One possibility is that bilateral coordination is more difficult than unilateral movements due to the greater degrees of freedom involved. A competing possibility is that movements of the intact limb entrain or inform movements of the residual muscles to be more consistent. Many regular activities, such as gait, involve movements of the body that are not coordinated in the modes explored in this study, and the application of the model-based control technique may not achieve the expected fidelity when the residual muscles must generate movements in a unilateral manner. Further study is required to determine both the real-world benefits of our approach and any potential improvements after a user training regimen.

Though this study verified the ability of the transtibial AMI to control a modeled subtalar joint specifically, potential translational application also exists at the transfemoral level. Consider an AMI for knee flexion and extension located within the transfemoral residuum and constructed from the residual biceps and rectus femoris. With slight modification of the neuromuscular modeling and optimization approach in this study, the transfemoral AMI may be used to control a modeled knee joint in free space with bandwidth matching that of an intact knee. By processing EMG from the user’s AMI as control input into this modeled knee, an online reference trajectory can be generated for a powered prosthetic knee. A feasible control paradigm embodiment then exists as a simple finite state machine, wherein swing portions of gait are volitionally determined by the online reference trajectory, and stance portions default to an impedance-based controller capable of gait speed adaption, such as the one tested by Kaveny et al. [60]. Advantages of volitional control during swing would include the immediate online adaptation of user gait, speed, and obstacle navigation for increased utility and safety.

## Conclusions

In this work, we developed a framework for quantifying the human bandwidth of lower-extremity movements by adapting rhythmic bilateral tests used in previous studies on upper-extremity movements. Using this framework, we provided additional evidence that motor coordination tendencies are relatively invariant across the different workspaces of the body, and that movement trajectories are formulated in task space coordinates independent of underlying biomechanical structures. Specifically, we demonstrated that transtibial AMI amputation subjects are able to control a neuromuscular model to produce subtalar kinematics similar to those produced by non-amputee subjects moving their intact subtalar joints, a task in which standard amputation subjects are unable to perform as well. This disparity in performance can be explained by the major difference between amputee populations, implicating that providing agonist-antagonist mechanoneural feedback is fundamental to the restoration of physiological motor control.

By demonstrating the ability of the AMI to generate biomimetic joint kinematics, new translational questions arise regarding current design criteria for neuroprosthetic devices. To be considered an ideal replacement, it is necessary for a biomimetic prosthesis design to meet or exceed the functional capability of the original biological limb with respect to both maximum torque and servo bandwidth. Though rough estimations of biological ankle movement bandwidth estimated from walking data have been used to guide the design of powered lower-extremity prostheses [61], few studies have analyzed lower-extremity joint bandwidth during free space movements. This deficiency is understandable from the perspective that restoration of locomotion is a primary design objective for powered lower-extremity prostheses, relegating the more complex feature of volitional control in free space to future research. However, given that the AMI is capable of biomimetic joint control, and because the results of this study also indicated a significant difference in bilateral coordination capability between intact ankle and subtalar joints, the authors propose that individual joint movement bandwidths should be characterized in further investigations and used as a mechatronic design objective for powered lower-extremity neuroprostheses capable of volitional control in free space. Doing so would facilitate the prosthesis user’s ability to navigate challenging terrain and achieve natural human behaviors such as foot tapping. Whatever the specific implementation details, it is our hope that consideration of this class of volitional movements in free space furthers the ideal of lower-extremity prosthesis embodiment.

## Supplementary information

**Additional file 1.** Supplementary figures and tables.

**Additional file 2.** Movie of sagittal condition ankle movement by non-amputee subject.

**Additional file 3.** Movie of symmetry condition subtalar movement by non-amputee subject.

**Additional file 4.** Movie of parallel condition subtalar movement by non-amputee subject.

## Data Availability

Original kinematic and sEMG data are protected under MIT Committee on the Use of Humans as Experimental Subjects (COUHES) protocol #1906898371, but may be available upon reasonable request. Other data are available in the main text and Additional file 1.

## Abbreviations

AMI: Agonist-antagonist myoneural interface
sEMG: Surface electromyography
LGNB: Lognormal distribution with bounded support
TA: Tibialis anterior
LGAS: Lateral gastrocnemius
TP: Tibialis posterior
PL: Peroneus longus
DP: Dorsiflexion-plantarflexion
IE: Inversion-eversion
IIR: Infinite impulse response
MTU: Muscle tendon unit
GA: Genetic algorithm
SD: Standard deviation
COUHES: Committee on the Use of Humans as Experimental Subjects
