# Correction to: Restoration of bilateral motor coordination from preserved agonist-antagonist coupling in amputation musculature

**DOI:** 10.1186/s12984-021-00846-y

**Published:** 2021-05-12

**Authors:** Tony Shu, Shan Shan Huang, Christopher Shallal, Hugh M. Herr

**Affiliations:** 1 MIT Media Lab, 75 Amherst St, Cambridge, MA 02139 USA; 2 Department of Mechanical Engineering, MIT, 77 Massachusetts Ave, Cambridge, MA 02139 USA; 3 Department of Biomedical Engineering, Johns Hopkins University, 720 Rutland Ave, Baltimore, MD 21205 USA; 4 Physical Medicine and Rehabilitation, Harvard Medical School, 25 Shattuck Street, Boston, MA 02115 USA

## Correction to: J NeuroEngineering Rehabil (2021) 18: 38 10.1186/s12984-021-00829-z

The original article contained two errors whereby both equation 1 and supplementary files were presented incorrectly.

The original article has since been updated to correct these errors [1].
