# Supplementary material for: Restoration of bilateral motor coordination from preserved agonist-antagonist coupling in amputation musculature
Source: J Neuroeng Rehabil. 2021 Feb 17;18:38. doi: 10.1186/s12984-021-00829-z (PMC7891024; doi:10.1186/s12984-021-00829-z)
Supplement: Supplementary file 1 — Additional file 1. Supplementary figures and tables. [file 12984_2021_829_MOESM1_ESM.pdf]

Supplementary Information for

Restoration of bilateral motor coordination from preserved agonist-antagonist coupling in amputation musculature

Tony Shu, Shan Shan Huang, Christopher Shallal, Hugh M. Herr

Correspondance to Hugh M. Herr.
E-mail: hherr@media.mit.edu

This PDF file includes:

Figs. S1 to S3
Tables S1 to S3
SI References

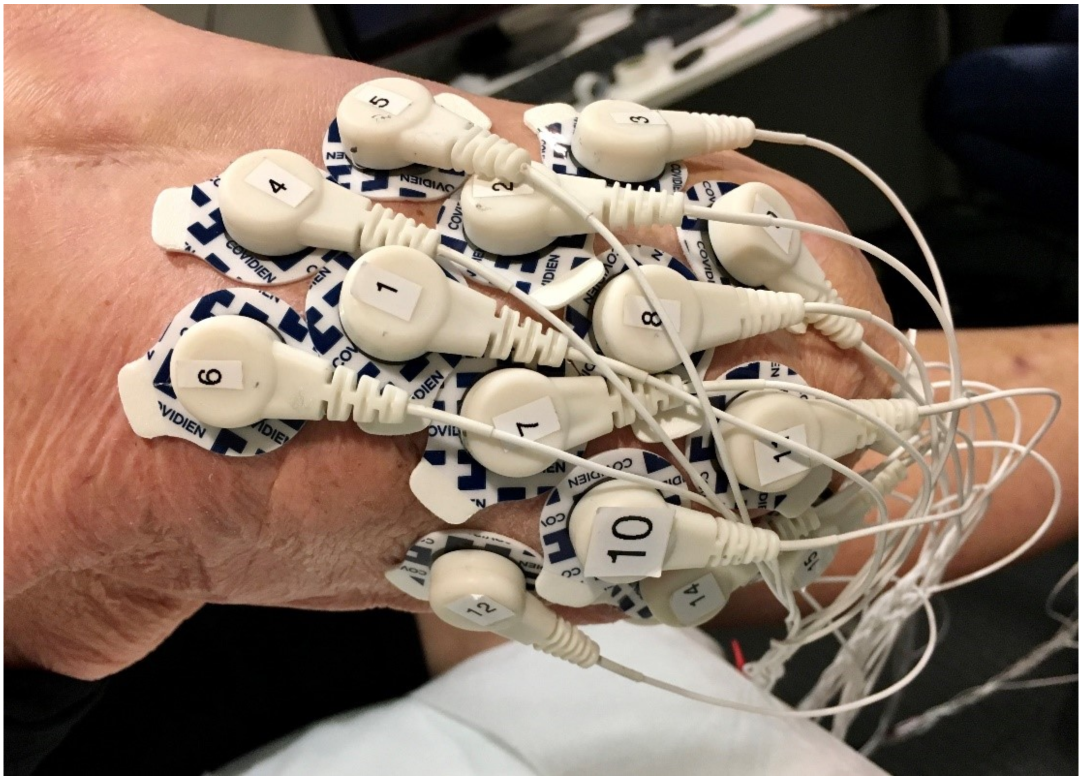

Fig. S1. Clusters of wet surface electrodes placed over lateral AMI muscles of the right transtibial residuum. Corresponding antagonist muscles are located medially on the residuum and correspondingly covered with electrodes.

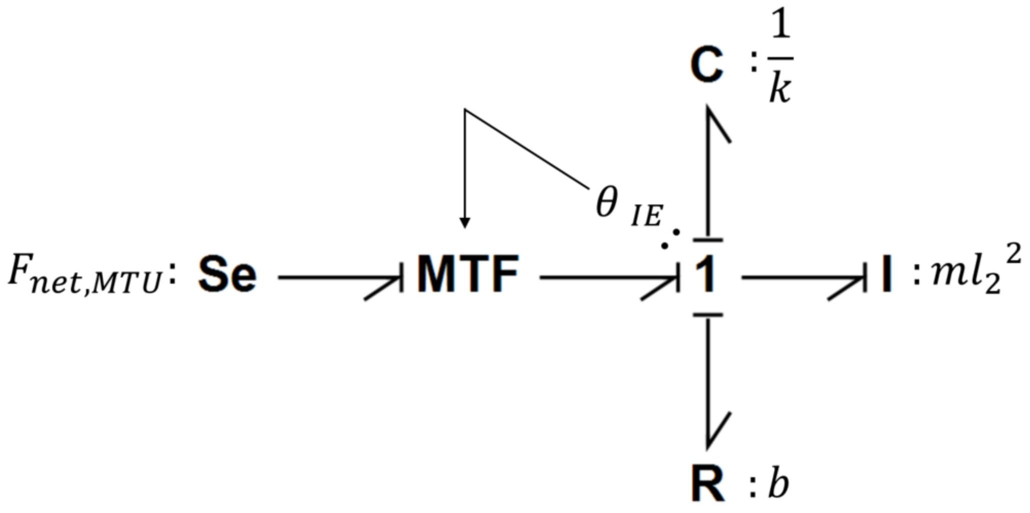

Fig. S2. Bond graph of neuromuscular subtalar model outlining energy flow. Net force contribution from the MTUs $F_{net,MTU}$ upon the variable moment arm acting as a modulated transformer (MTF) is modeled as an effort source into the second-order rotary system with damping (b) and stiffness (k). The structure of the energy storage elements allows for oscillatory behavior corresponding to rhythmic motions.

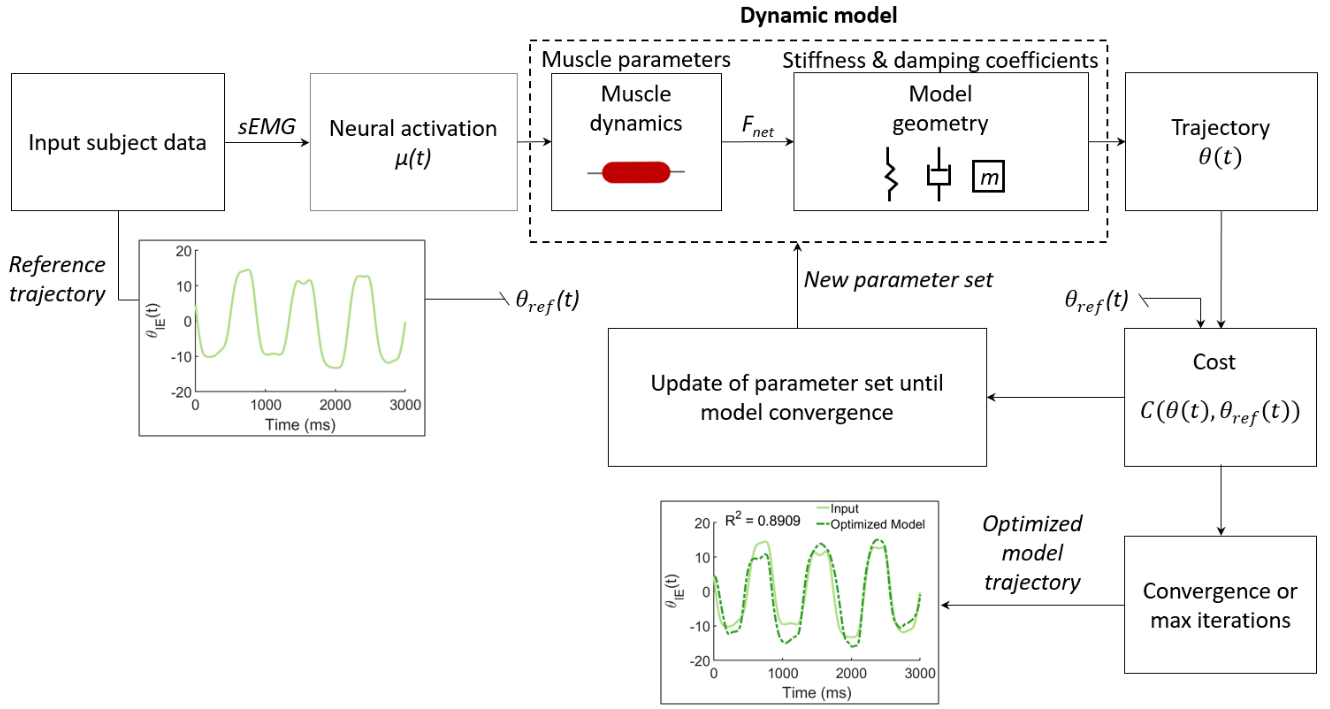

Fig. S3. Neuromuscular parameter optimization process. Given a three-second kinematic reference and neural excitation trajectory from a subject, the genetic algorithm optimization process identifies the set of model parameters across the search space which minimizes the kinematic trajectory's sum of squares error.

Table S1. Optimization parameter ranges and initial values

Parameter	Range	Initial Value
F_{max}	$0.05F_{max,0} \leq F_{max} \leq 6.00F_{max}$	$800.00N$
l_{opt}	$0.50l_{opt,0} \leq l_{opt} \leq 1.40l_{opt}$	$0.45m$
l_{slack}	$0.50l_{slack,0} \leq l_{slack} \leq 1.40l_{slack}$	$0.40m$
v_{max}	$7.00 \leq v_{max} \leq 12.00$	$10.00l_{opt}s^{-1}$
K_{active}	$0.10 \leq K_{active} \leq 0.70$	0.50
$K_{passive}$	$2.00 \leq K_{passive} \leq 7.00$	4.00
A_f	$0.01 \leq A_f \leq 0.70$	0.30
τ_{act}	$0.01 \leq \tau_{act} \leq 0.08$	$0.01s$
τ_{deact}	$0.03 \leq \tau_{deact} \leq 0.08$	$0.04s$
k	$0.00 \leq k \leq 15.00$	$0.00Nm\theta^{-1}$
b	$0.00 \leq b \leq 2.00$	$0.00Nm\dot{\theta}^{-1}$
m	constant	$0.40kg$
l_1	constant	$0.70m$
l_2	constant	$0.20m$
l_3	constant	$0.10m$

Table S2. Time-normalized velocity profile parameters from 1.4 Hz movements

Parameter	Physically Intact 1.4 Hz	ST Intact 1.4 Hz	ST Model 1.4 Hz	AMI Intact 1.4 Hz	AMI Model 1.4 Hz
Mean Peak Velocity (degrees/s)	152.05 ± 37.07	82.95 ± 34.83	90.88 ± 62.39	109.50 ± 39.30	114.77 ± 33.45
Skewness	-0.17 ± 0.21	-0.10 ± 0.26	-0.13 ± 0.59	-0.19 ± 0.20	-0.04 ± 0.32
Kurtosis	1.67 ± 0.16	1.66 ± 0.15	2.05 ± 0.69	1.70 ± 0.20	1.81 ± 0.30
R^2 LGNB	0.999	0.999	0.998	0.999	0.994

Parameters are presented as mean ± 1 SD where applicable. Though metrics are shared, the experimental conditions between Michmizos *et al.* (1) and this study are fundamentally different, and calculated metrics for the time-normalized velocity profiles provided in are for reference rather than direct comparison.

Table S3. Time-normalized velocity profile parameters from 2.2 Hz movements

Parameter	Physically Intact 2.2 Hz	ST Intact 2.2 Hz	ST Model 2.2 Hz	AMI Intact 2.2 Hz	AMI Model 2.2 Hz
Mean Peak Velocity (degrees/s)	124.02 ± 38.89	77.91 ± 35.36	75.13 ± 55.07	100.13 ± 35.70	91.14 ± 44.07
Skewness	-0.48 ± 0.15	-0.38 ± 0.22	-0.49 ± 0.43	-0.44 ± 0.19	-0.52 ± 0.15
Kurtosis	1.95 ± 0.18	1.88 ± 0.23	2.11 ± 0.63	1.91 ± 0.22	2.27 ± 0.75
R^2 LGNB	0.999	0.999	0.998	0.999	0.997

Parameters are presented as mean ± 1 SD where applicable. Though metrics are shared, the experimental conditions between Michmizos *et al.* (1) and this study are fundamentally different, and calculated metrics for the time-normalized velocity profiles provided in are for reference rather than direct comparison.

References

1. P Michmizos, L Vaisman, H Krebs, A comparative analysis of speed profile models for ankle pointing movements: Evidence that lower and upper extremity discrete movements are controlled by a single invariant strategy. *Front. Hum. Neurosci.* **8**, 962 (2014).
